# Cardiomyocyte Specific Deletion of ADAR1 Causes Severe Cardiac Dysfunction and Increased Lethality

**DOI:** 10.3389/fcvm.2020.00030

**Published:** 2020-03-18

**Authors:** Hamid el Azzouzi, Andreia P. Vilaça, Dries A. M. Feyen, Willemijn M. Gommans, Roel A. de Weger, Pieter A. F. Doevendans, Joost P. G. Sluijter

**Affiliations:** ^1^Laboratory of Experimental Cardiology, Circulatory Health Laboratory, Department of Cardiology, Regenerative Medicine Center, University Medical Center Utrecht, Utrecht, Netherlands; ^2^Department of Molecular Genetics, Erasmus University Medical Center, Rotterdam, Netherlands; ^3^Department of Biological Sciences, Lehigh University, Bethlehem, PA, United States; ^4^Department of Pathology, University Medical Center Utrecht, Utrecht, Netherlands; ^5^Interuniversity Cardiology Institute Netherlands, Royal Netherlands Academy of Sciences, Utrecht, Netherlands; ^6^Utrecht University, Utrecht, Netherlands

**Keywords:** UPR (unfolded protein response), microRNA, editing, apoptosis, heart failure

## Abstract

**Background:** Adenosine deaminase acting on RNA 1 (ADAR1) is a double-stranded RNA-editing enzyme that is involved in several functions including the deamination of adenosine to inosine, RNA interference (RNAi) mechanisms and microRNA (miRNA) processing, rendering ADAR1 essential for life.

**Methods and Results:** To investigate whether maintenance of ADAR1 expression is required for normal myocardial homeostasis, we bypassed the early embryonic lethality of ADAR1-null mice through the use of a tamoxifen-inducible Cre recombinase under the control of the cardiac-specific α-myosin heavy chain promoter (αMHC). Targeted ADAR1 deletion in adult mice caused a significant increase in lethality accompanied by severe ventricular remodeling and quick and spontaneous cardiac dysfunction, induction of stress markers and overall reduced expression of miRNAs. Administration of a selective inhibitor of the unfolded protein response (UPR) stress significantly blunted the deleterious effects and improved cardiac function thereby prolonging animal survival. *In vitro* restoring miR-199a-5p levels in cardiomyocytes lacking ADAR1 diminished UPR activation and concomitant apoptosis.

**Conclusions:** Our findings demonstrate an essential role for ADAR1 in cardiomyocyte survival and maintenance of cardiac function through a mechanism that integrates ADAR1 dependent miRNA processing and the suppression of UPR stress.

## Introduction

With a prevalence of over 23 million worldwide and increasing, heart failure is a leading public health burden, while its 5-year mortality rate of 50% is more than that of many cancers (1). Due to the multiple etiologies of heart failure, mechanisms that underlie this detrimental disease still remain to be clarified. Although regulation of cellular processes at transcriptional and post-translational levels is increasingly being explored, mechanisms underlying the post-transcriptional regulation in heart failure remains poorly understood. Essential players that are responsible for post-transcriptional regulatory control in eukaryotic organisms are the RNA binding proteins (RBPs).

RNA-binding proteins (RBPs) are a highly conserved, diverse, and significant part of the eukaryotic genome and have been implicated in every step of RNA synthesis and processing, including alternative splicing, RNA modification, polyadenylation, mRNA export, mRNA localization, translation, and mRNA turnover (2, 3). Likewise, RBP defects have been correlated with cancers, neuropathies, and other forms of human disease (4–6). One family of RBPs, adenosine deaminases acting on RNA (ADARs), catalyze the most prevalent RNA-editing mechanism consisting of hydrolytic deamination on adenosine in RNA transcripts hereby converting adenosine to inosine (7). Three *ADAR* genes are found in mammals, which encode two active deaminases (*Adar1* and *Adar2*) and one inactive deaminase (*Adar3*) (7). The adenosine to inosine conversion on double stranded RNAs by ADARs leads to a post-transcriptional modification process that not only contributes to genomic and proteomic diversity but also provides additional mechanisms of gene regulation (8). Next to their deaminase activity, recent studies describe versatile roles for ADARs through a direct protein-protein interaction (9, 10). In contrast to ADAR2, ADAR1 knockout animals are not viable due to embryonic lethality via a massive and widespread apoptosis beyond 11.5-12 dpc (11, 12). Although these studies indicate the absolute necessity of ADAR1 in cell survival and life overall, mechanisms that underlie the ADAR1 knockout phenotype in mice is unknown. In humans, ADAR1 has been linked to certain human disorders such as microcephaly mental retardation disorder and dyschromatosis symmetrica hereditaria, a rare autosomal-dominant inherited skin pigmentation disease (13, 14). In cancer, discrepancies in expression of ADAR1 highly associate with tumor development and progression (15, 16). In an elegant study, Qiu and colleagues investigated the role of ADAR1 in the intestinal tract using a conditional knockout mouse model showing marked changes in intestinal differentiation, rapid proliferation of transient amplification cells and severe intestinal injury and inflammation (17). Moreover, *Lgr5* driven loss of ADAR1 induced endoplasmic (ER) stress that caused a rapid apoptosis and loss of actively cycling stem cells in the small intestine and colon. Despite the fact that ADAR1 is highly expressed in the fetal and adult heart, hardly anything is known about the function of ADAR1 in the heart (12, 18).

In this study, assessing the function of ADAR1 in the heart through cardiomyocyte specific deletion, we describe a vital role for ADAR1 in maintaining cardiac physiology. Cardiomyocyte specific deletion of ADAR1 yielded an excessive amount of cardiomyocyte loss that resulted in cardiac dysfunction and eventual lethality. Lack of ADAR1 led to activation of the UPR driven apoptotic response, hampering ER stress handling in cardiomyocytes. Inhibition of the UPR in the ADAR1 knockout hearts significantly reduced cardiomyocyte loss and restored survival of the animals due to improved cardiac function. Further analysis indicated disturbed miRNA processing in ADAR1 knockout hearts, resulting in reduced levels of miR-199a-5p that balances ER stress induced UPR. Taken together, our data suggest a novel mechanism that links ADAR1 dependent miRNA synthesis to counteract the ER stress induced UPR in the heart.

## Results

### Cardiomyocyte Specific Deletion of ADAR1 in the Adult Heart Causes Severe Cardiac Dysfunction and Increased Lethality

**Figure 1:**
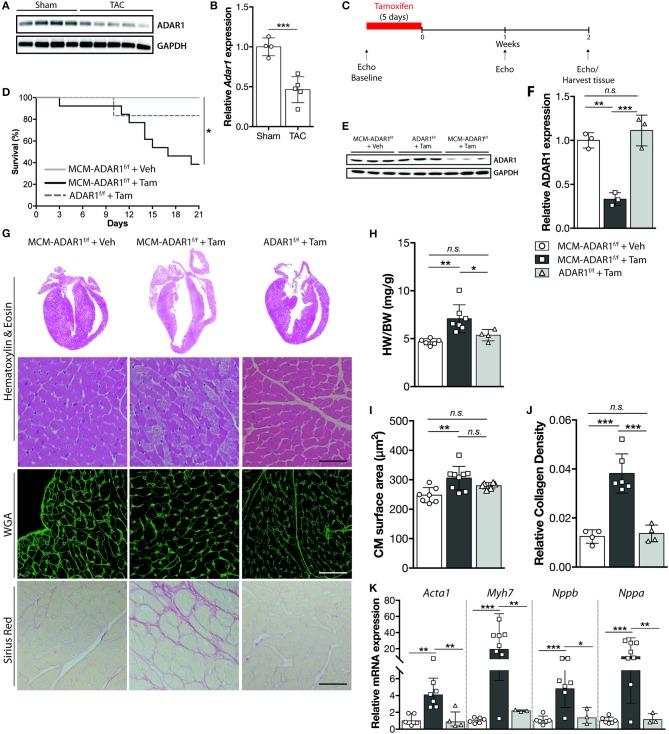
Cardiomyocyte specific ADAR1 deletion causes cardiomyocyte structural disintegration and lethality. **(A)** Western blot analysis of endogenous ADAR1 and GAPDH in sham or TAC operated mice. **(B)** Quantification of GAPDH-corrected protein levels of ADAR1 from **(A)**. **(C)** Design of tamoxifen-treatment study. **(D)** Kaplan-Meier survival curve of αMHC-MCM-ADAR1^F/F^ mice treated with vehicle (*n* = 11) or tamoxifen (*n* = 14) and ADAR1^F/F^ mice treated with tamoxifen (*n* = 6). **(E)** Western blot analysis of endogenous ADAR1 and GAPDH in vehicle or tamoxifen treated ADAR1^F/F^ and αMHC-MCM-ADAR1^F/F^ mice. **(F)** Quantification of GAPDH-corrected protein levels of ADAR1 from **(E)**. **(G)** Representative image of whole hearts (top panels), H&E-stained (middle panels), WGA labeled (middle panels), and Picroserious stained (lower panels) histological sections from ADAR1^F/F^ and αMHC-MCM-ADAR1^F/F^ mice treated with vehicle or tamoxifen. **(H)** Gravimetric analysis of corrected heart weights in ADAR1^F/F^ and αMHC-MCM-ADAR1^F/F^ mice, treated with vehicle or tamoxifen. **(I)** Analysis of cardiomyocyte hypertrophy in cardiac tissue sections. Slices (6 μm) of left ventricular myocardium were stained with Alexa 647–labeled wheat-germ agglutinin (WGA) for determination of myocyte cross-sectional areas. **(J)** Quantification of collagen density from. **(K)** Real-time PCR analysis of transcript abundance for fetal marker genes in hearts from ADAR1^F/F^ and αMHC-MCM-ADAR1^F/F^ mice, treated with vehicle or tamoxifen. *P < 0.05; **P < *0.01*, ***P < 0.005 vs. corresponding experimental group (error bars are s.e.m.).

Considering that, hardly anything is known about the function of ADAR1 in the healthy and failing heart, we first determined the levels of ADAR1 in failing murine hearts. Western blot analysis indicated decreased protein levels of ADAR1 in failing murine hearts after pressure overload by transverse aortic constriction (TAC) compared to sham operated mice (Figures 1A,B). Quantitative PCR analysis indicated that although *Adar1* transcript levels were maintained during the first 4 weeks of TAC, a significant decrease was shown in the decompensated phase of TAC induced heart failure (Supplemental Figure 1A). To investigate whether maintenance of *Adar1* expression is required for normal myocardial homeostasis and to bypass the early embryonic lethality of ADAR1-null mice, we triggered deletion of a floxed *Adar1* (ADAR1^F/F^) allele using a tamoxifen-inducible Cre recombinase under the control of the cardiac-specific α-myosin heavy chain promoter (αMHC) (Figure 1C). We forced *Adar1* gene deletion at the age of 12 weeks and noted that within two weeks after tamoxifen delivery, αMHC-MCM-ADAR1^F/F^ mice displayed signs of inactivity and a weak condition, as compared to all control groups. Indeed up to 60% of tamoxifen-treated αMHC-MCM-ADAR1^F/F^ mice died within 3 weeks after initiation of the treatment (Figure 1D). Specificity of *Adar1* gene deletion was shown by western blot analysis, demonstrating that our genetic intervention did induce a significant down regulation of ADAR1 protein levels in the αMHC-MCM-ADAR1^F/F^ mice treated with tamoxifen and not in the control groups, including vehicle treated αMHC-MCM-ADAR1^F/F^ or tamoxifen treated ADAR1^F/F^ mice (Figures 1E,F). Upon autopsy, tamoxifen-treated αMHC-MCM-ADAR1^F/F^ displayed severely enlarged hearts compared to control groups as indicated by an increase in heart weight body weight ratios (Figures 1G,H). Furthermore, at the individual cardiomyocyte level, FITC-labeled wheat-germ-agglutinin (WGA) staining showed a significant hypertrophic response in hearts of tamoxifen-treated αMHC-MCM-ADAR1^F/F^ mice (Figures 1G,I). Likewise, hearts lacking ADAR1 showed severe myocyte disarray that is accompanied with a significant increase of interstitial fibrosis (Figures 1G,J), revealing intricate features of clinical heart failure including potent re-activation of stress-induced genes such as *Acta1, Nppb, Myh7*, and *Nppa* in the tamoxifen-treated αMHC-MCM-ADAR1^F/F^ mice (Figure 1K).

**Figure 2:**
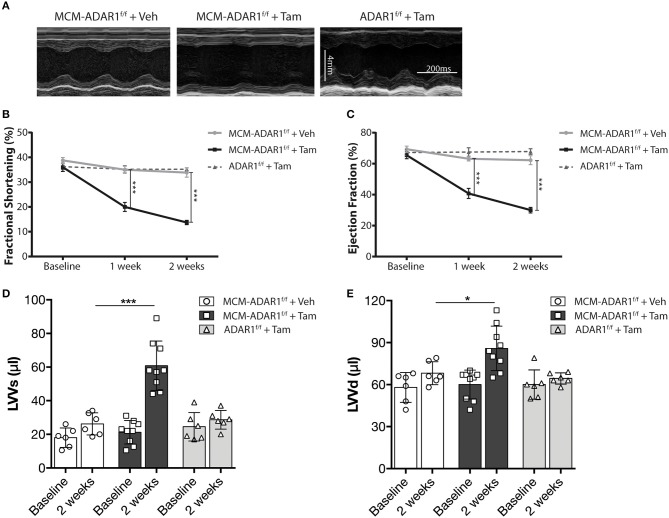
Conditional ADAR1 gene deletion promotes cardiac dysfunction. **(A)** Representative M-mode images from ADAR1^F/F^ and αMHC-MCM-ADAR1^F/F^ mouse hearts, treated with vehicle or tamoxifen. **(B)** Quantification of fractional shortening (FS), **(C)** ejection fraction (EF), **(D)** LV volume at systole (LVVs), and **(E)** LV volume at diastole (LVVd) by echocardiography in ADAR1^F/F^ and αMHC-MCM-ADAR1^F/F^ mouse, treated with vehicle or tamoxifen. *P < 0.05; ***P < 0.005 vs. corresponding experimental group (error bars are s.e.m.).

Cardiac geometry and function was assessed non-invasively by echocardiography at 1 and 2 weeks after tamoxifen treatment (Figure 2A, Supplemental Table 1). At these time points, tamoxifen-treated αMHC-MCM-ADAR1^F/F^ animals demonstrated a significant decline in cardiac contractility as evidenced by a massive decrease in fractional shortening (FS) and ejection fraction (EF) after 2 weeks (Figures 2B,C). Furthermore, tamoxifen-treated αMHC-MCM-ADAR1^F/F^ hearts showed a severe increase in left ventricular dimensions (Figures 2D,E), evidencing a rapid dilation of these hearts after deletion of ADAR1. These data demonstrate that conditional cardiac-specific deletion of ADAR1 in the adult heart causes rapid cardiac remodeling which results in multiple clinical signs of end-stage heart failure.

### Cardiomyocyte Specific Deletion of ADAR1 in the Adult Heart Leads to Cardiomyocyte Apoptosis via UPR Activation

**Figure 3:**
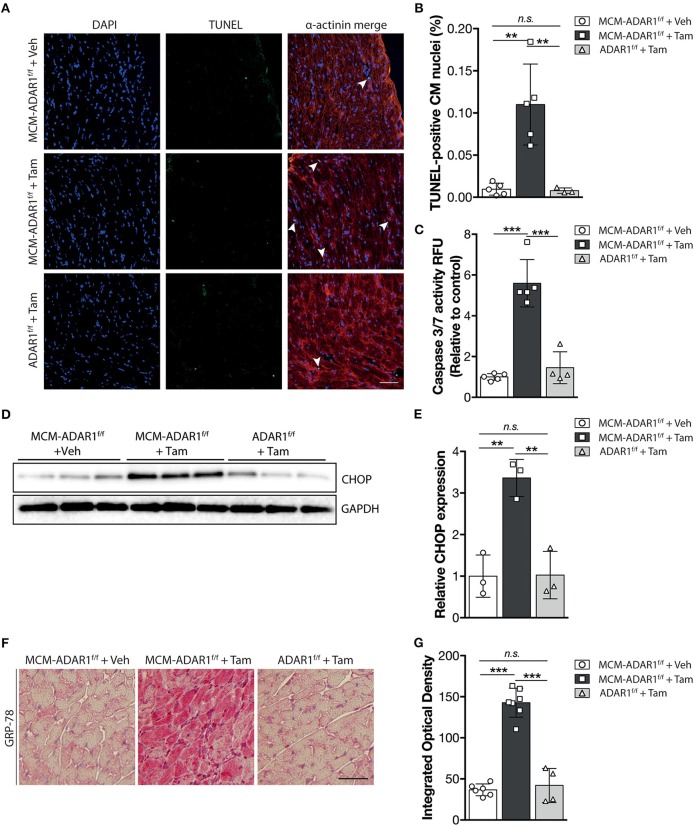
Targeted deletion of ADAR1 in the adult heart leads to activation of the UPR and cardiomyocyte apoptosis. **(A)** Representative images of TUNEL labeling from ADAR1^F/F^ and αMHC-MCM-AADR1^F/F^ mouse hearts, treated with vehicle or tamoxifen. **(B)** Bar graph indicating mean ± S.E. (error bars) of the percentage of TUNEL-positive cardiomyocytes from ADAR1^F/F^ and αMHC-MCM-ADAR1^F/F^ mouse hearts, treated with vehicle or tamoxifen. **(C)** Fluorometric assay measurement of Caspase-3/7 activity of ADAR1^F/F^ and αMHC-MCM-ADAR1^F/F^ mouse hearts lysates, treated with vehicle or tamoxifen. **(D)** Western blot analysis of endogenous CHOP and GAPDH in ADAR1^F/F^ and αMHC-MCM-ADAR1^F/F^ mouse hearts, treated with vehicle or tamoxifen. **(E)** Quantification of GAPDH-corrected protein levels of CHOP. **(F)** Immunohistochemical staining of ER-stress associated protein GRP-78, **(G)** with bargraph quantification of the immunoreactivity (red) in myocardium of ADAR1^F/F^ and αMHC-MCM-ADAR1^F/F^ mouse hearts, treated with vehicle or tamoxifen. **P < 0.01, ***P < 0.005 vs. corresponding experimental group (error bars are s.e.m.).

Studies using ADAR1 general knockout strategies, ascribe a prominent function for ADAR1 in maintaining cell survival both at the embryonic level and in the adult stage, hereby suggesting that the heart failure phenotype might be caused by a loss of cardiomyoyctes in the tamoxifen-treated αMHC-MCM-ADAR1^F/F^ mice. Indeed, terminal deoxynucleotidyl transferase-mediated deoxyuridinetriphosphate nick end labeling (TUNEL) staining confirmed a strong increase in apoptotic cardiomyocytes after deletion of ADAR1 (Figures 3A,B). In addition, measurement of Caspase-3/7 activity in a fluorometric assay revealed elevated enzyme activity in the tamoxifen-treated αMHC-MCM-ADAR1^F/F^ hearts but not in control littermates (Figure 3C). ADAR1 related induction of apoptosis has been shown to be mediated trough increased ER stress (17), whereby prolonged ER stress leads to accumulation of unfolded or misfolded proteins in the ER lumen and the subsequent activation of the unfolded protein response (UPR) (19). Heart failure has been associated with loss of cardiomyocytes due to activated UPR (19). To assess whether there is a potential role for the UPR in the increased cardiomyocyte loss in hearts lacking ADAR1, we measured levels of C/EBP homologous protein (CHOP), a marker for the UPR. In contrast to tamoxifen treated ADAR1^F/F^ and vehicle treated αMHC-MCM-ADAR1^F/F^ mice, tamoxifen-treated αMHC-MCM-ADAR1^F/F^ mice hearts showed a significant increase in CHOP levels (Figures 3D,E). Immunohistochemical (IHC) assessment of another UPR marker, glucose-regulating peptide 78 (GRP-78), showed a strong and specific upregulation of GRP-78 in cardiomyocytes of tamoxifen-treated αMHC-MCM-ADAR1^F/F^ mice and not in cardiomyocytes of tamoxifen treated ADAR1^F/F^ or vehicle treated αMHC-MCM-ADAR1^F/F^ mice (Figures 3F,G). As a control, GRP78 levels indeed showed to be upregulated in cardiomyocytes that were in the ischemic region of LAD infarcted mice hearts compared to the less stressed remote region (Supplemental Figure 1B). These outcomes suggest that the severe cardiac dysfunction and increased lethality observed in mice with a targeted deletion of ADAR1 in the adult heart, results from massive cardiomyocyte loss that is mediated by an activated UPR.

### Inhibition of the UPR Restores Cardiac Function in Mice With a Cardiomyocyte Specific Deletion of ADAR1

**Figure 4:**
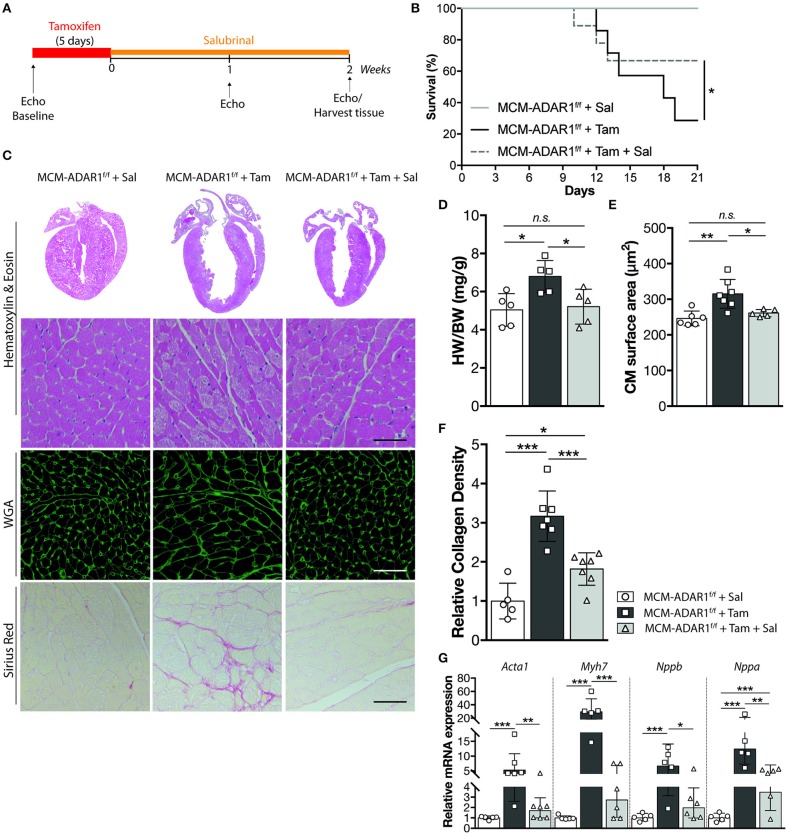
Inhibition of the UPR rescues from cardiomyocyte disintegration and lethality after deletion of ADAR1. **(A)** Design of Salubrinal-treatment study. **(B)** Kaplan-Meier survival curve of αMHC-MCM-ADAR1^F/F^ mice treated with Salubrinal (*n* = 9) or tamoxifen (*n* = 13) and αMHC-MCM-ADAR1^F/F^ mice treated with Tamoxifen and Salubrinal (*n* = 15). **(C)** Representative image of whole hearts (top panels), H&E-stained (middle panels), Alexa 647–labeled wheat-germ agglutinin (WGA) labeled (middle panels), and Picroserious stained (lower panels) histological sections from vehicle or tamoxifen treated αMHC-MCM-ADAR1^F/F^ mice with or without Salubrinal treatment. **(D)** Gravimetric analysis of corrected heart weights in vehicle or tamoxifen treated αMHC-MCM-ADAR1^F/F^ mice with or without Salubrinal treatment. **(E)** Analysis of cardiomyocyte hypertrophy in cardiac tissue sections for determination of myocyte cross-sectional areas. **(F)** Quantification of collagen density from. **(G)** Real-time PCR analysis of transcript abundance for fetal marker genes in hearts of vehicle or tamoxifen treated αMHC-MCM-ADAR1^F/F^ mice with or without Salubrinal treatment. *P < 0.05; **P < 0.01, ***P < 0.005 vs. corresponding experimental group (error bars are s.e.m.).

To directly investigate the role for the UPR in cardiac dysfunction caused by ADAR1 depletion, we administrated Salubrinal, an ER stress inhibitor (20), to tamoxifen-treated αMHC-MCM-ADAR1^F/F^ mice (Figure 4A). Interestingly, this selective inhibitor significantly blunted the deleterious effects seen in tamoxifen treated αMHC-MCM-ADAR1^F/F^ mice. Salubrinal administration rendered these mice more active and effectively reduced the mortality seen in tamoxifen treated αMHC-MCM-ADAR1^F/F^ mice (Figure 4B). Gross morphology of hearts of tamoxifen-treated αMHC-MCM-ADAR1^F/F^ mice that were co-treated with Salubrinal mice showed significant normalization of cardiac geometry as depicted by the heart weight body weight ratio (Figures 4C,D). In line, at the individual cardiomyocyte level there was a significant decrease of hypertrophy in hearts of tamoxifen-treated αMHC-MCM-ADAR1F/F mice that were co-treated with Salubrinal compared to the control counterparts (Figures 4E,G). Moreover, H&E stainings showed a strong restoration in myocyte integrity after treatment with Salubrinal (Figure 4C) that is supported by a decrease of interstitial fibrosis in hearts of tamoxifen-treated αMHC-MCM-ADAR1^F/F^ mice that were co-treated with Salubrinal (Figures 4C,F). This attenuation of cardiac remodeling by Salubrinal included the normalization of stress-induced genes, indicating prevention of end-stage heart failure of tamoxifen treated αMHC-MCM-ADAR1^F/F^ mice (Figure 4G).

**Figure 5:**
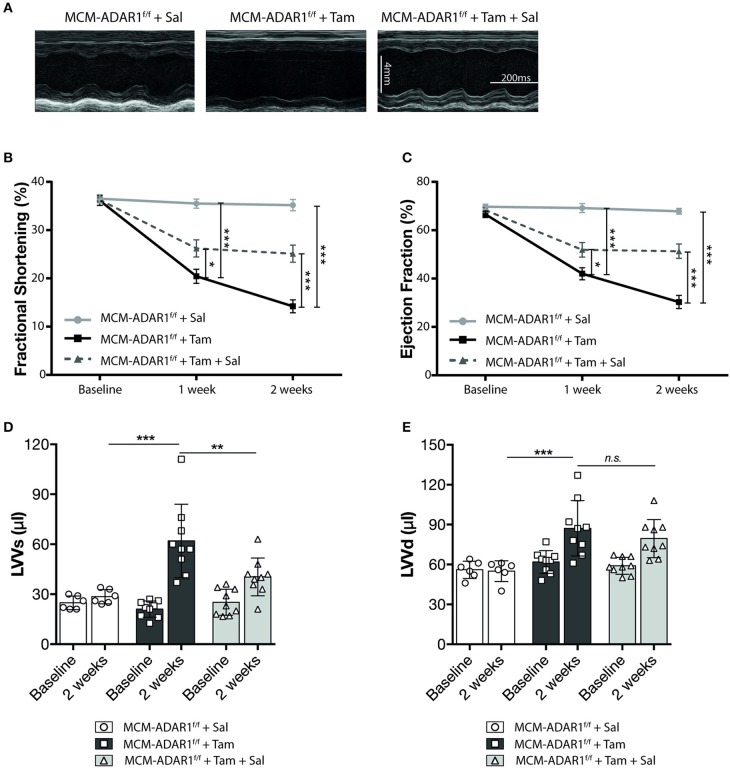
Inhibition of the UPR restores cardiac function after deletion of ADAR1. **(A)** Representative M-mode images from vehicle or tamoxifen treated αMHC-MCM-ADAR1^F/F^ mice with or without Salubrinal treatment. **(B)** Quantification of fractional shortening (FS), **(C)** ejection fraction (EF), **(D)** LV volume at systole (LVVs), and **(E)** LV volume at diastole (LVVd) by echocardiography in vehicle or tamoxifen treated αMHC-MCM-ADAR1^F/F^ mice with or without Salubrinal treatment. *P < 0.05; **P < 0.01, ***P < 0.005 vs. corresponding experimental group (error bars are s.e.m.).

**Figure 6:**
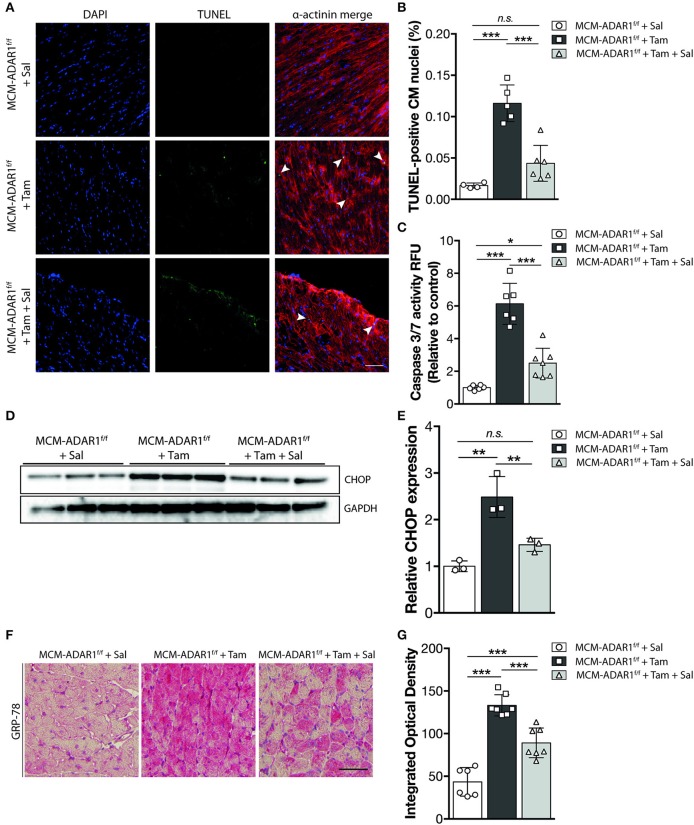
Salubrinal inhibits UPR driven cardiomoycte apoptosis in hearts lacking ADAR1. **(A)** Representative images of TUNEL labeling from αMHC-MCM-AADR1F/F mouse hearts, treated with the stated combination of Salubrinal and tamoxifen. **(B)** Bar graph indicating mean ± S.E. (error bars) of the percentage of TUNEL-positive cardiomyocytes from αMHC-MCM-AADR1F/F mouse hearts, treated with the stated combination of Salubrinal and tamoxifen. **(C)** Fluorometric assay measurement of Caspase-3/7 activity of αMHC-MCM-AADR1F/F mouse hearts lysates, treated with the stated combination of Salubrinal and tamoxifen. **(D)** Western blot analysis of endogenous CHOP and GAPDH in αMHC-MCM-AADR1F/F mouse hearts, treated with the stated combination of Salubrinal and tamoxifen **(E)** Quantification of GAPDH-corrected protein levels of CHOP. **(F)** Immunohistochemical staining of ER-stress associated protein GRP-78 with **(G)** bargraph quantification of the immunoreactivity (red) in myocardium of from αMHC-MCM-AADR1F/F mouse hearts, treated with the stated combination of Salubrinal and tamoxifen. *P < 0.05; **P < 0.01, ***P < 0.005 vs. corresponding experimental group (error bars are s.e.m.).

Furthermore, echocardiographical analysis at 1 and 2 weeks after tamoxifen treatment, demonstrated a significant recovery of cardiac contractility as evidenced by an increase in fractional shortening and ejection fraction only after Salubrinal treatment (Figures 5A–C, Supplemental Table 2). Normalization of the cardiac structure was also visible in left ventricular dimensions of tamoxifen-treated αMHC-MCM-ADAR1^F/F^ mice that were co-treated with Salubrinal (Figures 5D,E). Although Salubrinal did not fully restore the cardiac function of mice lacking cardiac ADAR1, these findings demonstrate that deterioration of cardiac function after ADAR1 deletion strongly correlates with the activation of the UPR mediated cardiac tissue loss in these hearts. Determination of cardiomyocyte apoptosis using TUNEL confirmed a significant reduction in TUNEL positive cardiomyocytes after treatment with Salubrinal (Figures 6A,B). Moreover, measurement of Caspase-3/7 activity in a fluorometric assay revealed elevated enzyme activity in the tamoxifen-treated αMHC-MCM-ADAR1^F/F^ hearts which was reversed after treatment with Salubrinal (Figure 6C). To determine whether the lowered cardiomyocyte loss in these animals coincides with an attenuation of the UPR, we evaluated the same UPR markers in these animals. Levels of CHOP were reduced in tamoxifen-treated αMHC-MCM-ADAR1^F/F^ mice that were co-treated with Salubrinal (Figures 6D,E). In addition, immunohistochemical assessment of GRP-78 showed a significant down-regulation in cardiomyocytes of tamoxifen-treated αMHC-MCM-ADAR1^F/F^ mice that received Salubrinal and not in the control groups (Figures 6F,G). These data indicate that effective inhibition of UPR suffices to partly prevent cardiomyocyte loss seen in mice hearts with a cardiomyocyte specific deletion of ADAR1, and thereby restore its function via prolonging cardiomyocyte survival.

### Global Reduction in miRNA Production After Cardiomyocyte Specific Deletion of ADAR1 in the Adult Heart Sources the UPR

**Figure 7:**
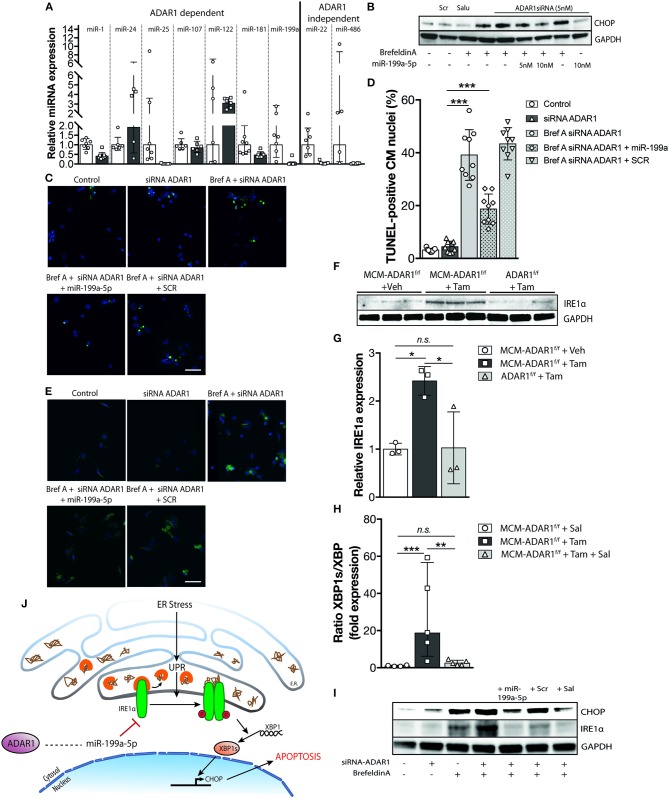
Global reduction in miRNA production after deletion of ADAR1 in the adult heart sources the UPR. **(A)** Real-time PCR analysis of transcript abundance for ADAR1 dependent miRNAs in hearts of vehicle or tamoxifen treated αMHC-MCM-ADAR1^F/F^ mice. **(B)** Western blot analysis of endogenous CHOP and GAPDH in neonatal rat cardiomyocytes transfected with miR-199a-5p mimicks and ADAR1 siRNA or scrambled miR for 48 h and treated with vehicle or Brefeldin A for 24 h. **(C)** Representative images of TUNEL labeling in neonatal rat cardiomyocytes treated with vehicle or Brefeldin A and transfected with miR-199a-5p and ADAR1 siRNA or scrambled miR. **(D)** Bar graph indicating mean ± S.E. (error bars) of the percentage of TUNEL-positive neonatal rat cardiomyocytes treated with vehicle or Brefeldin A and transfected with miR-199a-5p and ADAR1 siRNA or scrambled miR. **(E)** Immunofluorescence staining of ER-stress associated protein GRP-78 in neonatal rat cardiomyocytes treated with vehicle or Brefeldin A and transfected with miR-199a-5p mimicks and ADAR1 siRNA or scrambled miR. **(F)** Real-time PCR analysis of transcript abundance for *XBP-1* and *XBP-1s* in hearts of vehicle or tamoxifen treated αMHC-MCM-ADAR1^F/F^ mice with or without Salubrinal treatment. **(G)** Western blot analysis of endogenous IRE1α and GAPDH in ADAR1^F/F^ and αMHC-MCM-ADAR1^F/F^ mouse hearts, treated with vehicle or tamoxifen. **(H)** Quantification of GAPDH-corrected protein levels of IRE1α from **(G)**. **(I)** Western blot analysis of endogenous IRE1α and GAPDH in neonatal rat cardiomyocytes treated with vehicle or Brefeldin A and transfected with miR-199a-5p mimicks or and ADAR1 siRNA or scrambled miR. **(J)** Schematic representation of the proposed mechanism. *P < 0.05; **P < 0.01, ***P < 0.005 vs. corresponding experimental group (error bars are s.e.m.).

Recent studies have described a novel role of ADAR1 in promoting global miRNA production (10, 21). Total RNA analysis in mouse embryos lacking ADAR1 showed a massive decrease of miRNA reads compared to control wild-type embryos, probably underlying the lethality due to widespread apoptosis that is seen in these embryos (21). Moreover, ADAR1 was shown to directly interact with DICER in a RNA binding independent manner to promote processing of miRNAs, RISC loading of miRNAs, and the subsequent silencing of target RNAs (21). To confirm the reduced miRNA expression in hearts of tamoxifen-treated αMHC-MCM-ADAR1^F/F^ mice independently and more quantitatively, we determined the expression of previously described ADAR1 dependent and independent miRNAs. Quantitative PCR analysis indicated reduced levels of ADAR1 dependent miRNAs in hearts of tamoxifen-treated αMHC-MCM-ADAR1^F/F^ mice compared to vehicle treated mice (Figure 7A). Because ADAR1 dependent miRNA synthesis is crucial for development (10, 21), we hypothesized that the hampered miRNA synthesis underlies the cardiomyocyte loss seen in hearts of tamoxifen-treated αMHC-MCM-ADAR1^F/F^ mice. Already several studies have described miRNAs to constitute an additional layer in the regulation of the UPR in different physiological and pathophysiological contexts including cardiovascular diseases (22–24). To investigate whether ADAR1 dependent miRNAs regulate the UPR in cardiomyocytes, we tested several miRNAs that were downregulated in hearts of tamoxifen-treated αMHC-MCM-ADAR1^F/F^ mice. Isolated wild-type rat cardiomyocytes were transfected with an siRNA targeting ADAR1 and treated with Brefeldin A, a well-known inducer of the UPR (25) (Supplemental Figure 1C). Additionally, co-transfection with several candidate miRNAs that are also reported to have a role in UPR regulation proved miR-199a-5p to significantly attenuate UPR activation in cardiomyocytes (Supplemental Figure 1D). Moreover, cardiomoyctes that were transfected with miR-199a-5p showed to be more viable (Supplemental Figure 1E), with a reduced protein levels of CHOP after treatment with BrefeldinA, indicating resistance toward BrefeldinA induced activation of the UPR (Figure 7B). Immunofluorescent staining exhibited lower levels of GRP-78 in cardiomyocytes after transduction with miR-199a-5p that is nearly comparable to Salubrinal treatment (Figure 7E). Likewise, miR-199a-5p considerably decreased the number of TUNEL positive cardiomyocytes signifying a protective effect on UPR induced apoptosis in cardiomyocytes (Figures 7C,D).

At a molecular level, during UPR, increased binding of GRP-78 to misfolded proteins activates among others the inositol-requiring enzyme 1α (*Ire1*α), a dual-function protein with endoribonuclease and protein kinase activities. Activation of IRE1α is required for the splicing and activation of X-box-binding protein 1 (*Xbp-1*), leading to expression of several ER stress-responsive genes such as the pro-apoptotic transcription factor CHOP (26). In hepatocytes, miR-199a-5p was shown to directly target the *Ire1*α 3′UTR and exert a protective effect during hepatic ER stress (23, 27). To determine the level of activity of *Ire1*α in tamoxifen-treated αMHC-MCM-ADAR1^F/F^ mice, we performed quantitative PCR analysis to delineate the ratio of spliced X*bp-1* (*Xbp-1s*). Cardiac deletion of ADAR1 significantly increased levels of *xbp1s* compared to unspliced isoforms, indicating an increase in IRE1α activity in these hearts (Figure 7F). In addition, cardiac IRE1α protein levels were increased in tamoxifen-treated αMHC-MCM-ADAR1^F/F^ mice compared to control counterparts (Figures 7G,H). *In vitro*, expression of miR-199a-5p in cardiomyocytes showed a significant dose dependent inhibition of IRE1α, resulting in lower activation of CHOP (Figure 7I). Altogether, our data suggest an important role for ADAR1 in the heart, whereby ADAR1 balances the UPR and maintains cardiomyocyte integrity through regulation of miRNAs (Figure 7J).

## Discussion

Despite the increasing attention for the roles of RBPs, such as ADAR1, as several studies show striking effects in several human diseases, the role of ADAR1 in the heart is unexplored. Lessons learned from knockout strategies designate a key role for ADAR1 in maintaining viability and the likely involvement of ER stress in cell death triggered by ADAR1 deletion. Cardiomyocytes, compared to other cell types, strongly depend on the sarcoendoplasmatic reticulum (SER) for normal cellular homeostasis and contractility. Moreover, stress stimuli such as oxidative stress, hypoxia, and ischemic insult induce ER stress leading to heart failure (19). Here, we describe a mechanism that links ADAR1 function to ER stress induced cardiomyocyte loss, involving a unique ADAR1- dependent microRNA synthesis that balance the ER stress induced UPR, counter-balancing cardiomyocyte apoptosis. We demonstrate that cardiomyocyte specific deletion of ADAR1 results in an exaggerated decline in cardiac function and animal survival as a result of cardiomyocyte loss. In depth, these cardiomyocytes displayed a salient increase of ER stress induced UPR that sourced the cardiomyocyte loss. Moreover, compound induced inhibition of the UPR restored survival of cardiomyocytes that lack ADAR1 and restored overall cardiac function.

Although the majority of research on ADAR1 has focused on the deamination of adenosine to inosine in double-stranded RNAs, more recent studies substantiated other versatile roles for ADAR1. Studies on antiviral expression cascades demonstrated that interaction of ADAR1 with nuclear factor 90 (NF90) family proteins and the subsequent suppression of the interferon-β pathways protected hematopoietic progenitor cells from apoptosis (28). A more complex role is given to ADAR1 in the regulation of heterochromatin silencing. In this study, Wang et al., describe the involvement of ADAR1 in the vigilin/ADAR1/Ku heterodimer/RHA complex that recruits the DNA-protein kinase DNA-PKcs enzyme, which phosphorylates a set of target proteins such as HP1α and H2AX and also plays a main role in the chromatin silencing mechanism (29). Vigilin, as a cellular factor, binds to deaminated RNA and physically interacts with ADAR1, suggests other possible roles next to A → I RNA editing for ADAR1 in the heterochromatic gene-silencing mechanism. In an elegant study, Ota et al., demonstrated diverse roles for ADAR1 that were dependent on its complex partner. In contrast to the requirement of homodimerization for the A-to-I RNA editing activities of ADAR1 (30), dimerization with DICER via the second dsRBD of ADAR1 was necessary for miRNA production (21). The interaction with DICER was RNA binding independent and promoted processing of miRNAs, through RISC loading of miRNAs, leading to increased silencing of target RNAs. What more, complex formation of ADAR1 with DICER led to conformational changes in DICER that decreased its turnover rate resulting in an increase in the amount of miRNA processing. In line, mouse embryos lacking ADAR1 showed a suppression of specific miRNA production around E11-12 compared to wildtype (21). Knowing that RNAi deficiency in such short time frame results in embryonic lethality in ADAR1^−/−^, points to a more prominent role for ADAR1 in RNAi function rather than RNA editing in these embryos. Although the study of Ota et al., pointed out a differential expression pattern for miRNAs that presumably are regulated by the ADAR1/DICER complex, analysis of some of these miRNAs did not confirm this profile. For example, miRNAs miR-22 and miR-486, indicated to be ADAR1/DICER complex independent showed lower expression levels in the cardiomyocyte specific ADAR1 deleted hearts. Vice versa, ADAR1/DICER complex dependent miRNA's, miR-24 and miR-122, were upregulated in cardiomyocyte specific ADAR1 deleted hearts. This discrepancy could be attributed to the expression levels of these miRNAs in non-cardiomyocytes in the two hearts. In addition, Heale et al. (31) reported that ADAR2 has a role in modulating microRNAs by disrupting Drosha processing, indicating that ADARs regulate miRNA processing at multiple levels and that more research will be needed to understand how ADARs cooperates to change the level of specific miRNAs. Conversely, although we cannot rule additional influences in the tamoxifen-treated αMHC-MCM-ADAR1^F/F^ mice, suppressed miRNA levels most likely underlies the phenotype of these hearts. In our study, reinstalling miR-199a-5p in cardiomyoctes after knocking down ADAR1 prevented ER stress induced cell death, underlining the significance of functional miRNAs in this setting. However, to rule out any biological effects of Cre expression in the various cell types, tamoxifen treated *aMHC-MCM-Adar1*^+/+^ mice would have been an optimal control.

Only recently, the relevance for functional miRNA processing in ER stress has been described (22, 32). While in its infancy, research on how miRNA impact mechanisms that underlie the adaptive/apoptotic UPR-switch clearly indicate a crucial part for miRNAs in regulating the complex UPR signaling. Though the evolutionary conserved UPR is initially an adaptive response that mainly functions to compensate and restore organelle equilibrium, prolonged ER stress eventually leads to cell death. The UPR is composed of three main signaling pathways including activating transcription factor-6 (ATF6), PKR-like eukaryotic initiation factor 2 kinase (PERK) and inositol-requiring enzyme-1 (IRE-1) (19). ATF6 is induced under ischemic conditions and has been described to have role in the adaptive UPR response in the heart as inhibition of ATF6 resulted in impaired cardiac function and increased mortality (19, 33). In contrast, prolonged activation of the PERK/ATF4/CHOP pathway has been proven to induce translational arrest and cardiac cell death (34). In line, CHOP deficiency has been shown to reduce myocardial reperfusion injury in a mouse model of myocardial infarction (35). In our study, increased cardiomycyte loss in the tamoxifen-treated αMHC-MCM-ADAR1^F/F^ mice was accompanied with augmented protein levels of CHOP which was reversed after attenuation of the ER stress induced UPR in these mice. During ER stress, the ER transmembrane protein IRE-1α is homodimerized causing its autophosphorylation and activation of its endoribonuclease to splice XBP-1 mRNA (36). Though XBP-1 is indispensable for heart formation and XBP-1 knockout mice show significant higher levels of cardiomyocyte death, XBP-1s induces ER stress response genes that escape PERK-mediated translational arrest (36, 37). In this study, miR-199a-5p effectively targets IRE-1α indicating that the protective role of this miRNA is due to the lowering of the endoribonuclease activity leading to lower levels of XBP-1s. In fact, quantitative PCR analysis showed no effects on *Xbp-1* levels in all the αMHC-MCM-ADAR1^F/F^ mice groups but significantly lowered *Xbp-1s* levels after treatment with Salubrinal, substantiating the role of XBP-1s in cardiomyocte death after cardiac deletion of ADAR1. In general, miR-199a-5p is a multifaceted miR that is shown to regulate cell survival and metabolism in different heart failure models (38, 39). As the initial phases of the UPR are set out to prevent further translational loading of the ER by attenuating translation and cell cycle arrest, regulation of the adaptive UPR response is probably sustained at the level of miRNAs. Like in muscle, continuous contraction requires a well-balanced calcium handling by the sarcoplasmic reticulum. Perturbations in this equilibrium can cause exaggerated effects tilting the UPR-switch toward an apoptotic outcome (40–42). In this setting, it remains interesting how ADAR1 function is regulated during the UPR and the role of other ADAR1 dependent miRNAs in fine tuning the delicate balance of the adaptive/apoptotic UPR-switch. Although this study mainly focused on the role of ADAR1 in cardiomyocytes, it would be worthwhile to investigate this also in the other cardiac cell types. Expression levels of ADAR1 in non-cardiomyocytes has been confirmed in studies as well as relevance of UPR regulation. How the two intricate in these cells could help us understand UPR related pathology.

As the significance of ADAR1 in both physiological and pathological conditions is only now starting to be explored, is becoming clear that ADAR1 centers a complex equilibrium composed of several layers of regulation comprising editing events, post-translational modifications and homo-hetero-dimer formation. Already signified by several studies, clarifying the function of ADAR1 requires a multicenter approaches including tissue specific depletion or up-regulation of ADAR1. To our knowledge, for the first time our data signify ADAR1 to be vital for maintaining cardiac homeostasis and function in the adult heart via an intricate mechanism involving miRNA and ER stress handling. Disturbance of this process in the heart due to cardiomyocyte specific deletion of ADAR1 results in exaggerated cell death and severe decline of overall cardiac function eventually culminating in heart failure and increased mortality. Although these hearts showed increased cell death (±0.1%), compared to a rate of 0.03% in human-dilated cardiomyopathy, this resulted in a more interstitial fibrotic response whereas loss of tissue at this rate would initiate replacement fibrosis. It remains fascinating whether this initial response would eventually culminate into replacement fibrosis as a reaction to continued tissue loss in hearts lacking ADAR1.

In addition, although the role of ADAR1 in calcium handling is not known, ADAR1 editing of GRIK1 and GRIK2 has been shown to affect calcium channel permeability (43). Lack of ADAR1 could therefore theoretically sensitize cardiomyocytes toward calcium mishandling and arrhythmia. However, no signs of arrhythmia were seen in these mice during echocardiographic analysis.

This study proposes a mechanism that underlies the role in cell survival for ADAR1 and its relevant regulation of the UPR that hallmarks heart failure. Despite the strong effect of ADAR1 knockdown on cardiac homeostasis it is still a daunting task to elucidate it's exact role taking into account the multitude of regulation that is governed through ADAR1. Further understanding the mechanisms that maintain this balance and the role played by RNA binding proteins such as ADARs in overall cardiac physiology is not only crucial but will aid innovative therapeutic intervention.

## Experimental Procedures

### Mouse Models

ADAR1^F/F^ mice, described in Wang et al. (12), were purchased from the Jackson Laboratories (B6.129-ADAR^*tm*1*Knk*^). Mice harboring a tamoxifen-regulated form of Cre recombinase (MerCreMer) under control of the murine *Myh6* promoter (αMHC-MerCreMer; MCM mice) (44) in a B6129F1 background, were used to generate αMHC-MCM-ADAR1^F/F^ mice. ADAR1^F/F^ and αMHC-MCM-ADAR1^F/F^(mixed genders) were treated with either vehicle (10/90 v/v ethanol/peanut oil, Sigma P2144) or tamoxifen (45 mg/kg per day) by daily intraperitoneal (IP) injections for 5 consecutive days. Furthermore, in the Salubrinal treated group, mice received IP injections of salubrinal [1 mg/kg body weight, Salubrinal (Tocris #2347)] for 14 days after tamoxifen injections. Salubrinal was dissolved in DMSO and then in saline. The control group was IP injected with equal volume of DMSO and saline. Adult αMHC-MCM-ADAR1^F/F^ mice and ADAR1^F/F^ mice (10–12 weeks of age) were used for functional and histological analyses. All experiments were carried out in accordance with the *Guide for the Care and Use of Laboratory Animals*, with prior approval by the Animal Ethical Experimentation Committee, Utrecht University. Investigators were blinded to treatment for physiological and biochemical measurements.

### Transthoracic Echocardiography

For echocardiography, mice were shaved and lightly anesthetized with isoflurane (mean 1.5% in oxygen) and allowed to breathe spontaneously via a nasal cone. Non-invasive, echocardiographic parameters were measured using a Vevo®-2100 high frequency ultrasound system (VisualSonics Inc., Toronto, Canada) and analyzed with Vevo®-2100-1.6.0 software. Long-axis EKG-triggered cine loops of the left ventricular (LV) contraction cycle were obtained in B-mode to assess end-diastolic/systolic volume. Short-axis recordings of the LV contraction cycle were taken in M-mode to assess wall thickness of the anterior/posterior wall at the mid-papillary level. From B-mode recordings, LV length from basis to apex, LV volumes in systole (LVVs) and diastole (LVVd) were determined. From M-mode recordings, LV posterior wall thickness in systole (LV PWs) and diastole (LV PWd) were determined. LV mass was calculated with the following formula: (0,8^*^(1.04^*^(((LVIDd + LV PWd + IVSd)∧3)-((LVIDd)∧3))+0,6); fractional shortening (FS) was calculated with the following formula: (LVIDd-LVIDs)/LVIDd^*^100). Ejection fraction (EF) was calculated as ((SV/Vd)^*^100) with Vs, systolic volume (3,1416^*^(LVIDs∧3)/6), Vd, diastolic volume (3,1416^*^(LVIDd∧3)/6), and SV, stroke volume (Vd-Vs).

### (microRNA/mRNA) Real-Time PCR

Primer sequences used are listed in Table 1. TRIzol reagent (Invitrogen) was used to isolate RNA from mouse or human tissue followed by reverse-transcription reaction with either Superscript II reverse transcriptase (Invitrogen) or for miRNA transcript detection with miScript Reverse Transcription Kit (Qiagen). Real-time PCR was performed using miScript SYBR Green PCR Kit (Qiagen), on a BioRad iCycler (Biorad). To determine transcript quantity, the relative Ct method was used, where the amount of target normalized to the amount of endogenous control (U6) and relative to the control sample is given by 2^−ΔΔCt^.

**Table 1:** Real-time PCR primers used in this study.

**Gene**	**Forward primer**	**Reverse primer**
*Acta1*	5′-CCGGGAGAAGATGACTCAAA	5′-GTAGTACGGCCGGAAGCATA
*Adar1*	5′-CCACCTCCAGTGCGGAGTAGCG	5′TGCCCCTTGAGAAATTCTATTTGC
*Myh7*	5′-CGGACCTTGGAAGACCAGAT	5′-GACAGCTCCCCATTCTCTGT
*Nppa*	5′-TCTTCCTCGTCTTGGCCTTT	5′-CCAGGTGGTCTAGCAGGTTC
*Nppb*	5′-TGGGAGGTCACTCCTATCCT	5′-GGCCATTTCCTCCGACTTT
*Xbp-1*	5′-ACATCTTCCCATGGACTCTG	5′-TAGGTCCTTCTGGGTAGACC
*Xbp-1s*	5′-TGCTGAGTCCGCAGCAGGTG	5′-GCTGGCAGGCTCTGGGGAAG
*L7*	5′-CGAGAAAAAGGCCCGCAAGG	5′-GCTTGACGAACACTCCGTTG

*All oligo's are depicted in 5′ –> 3′direction*.

### Primary Cardiomyocytes Isolation, Transient Transfections

Neonatal rat cardiomyocytes isolation was performed with Pierce Primary Cardiomyocyte Isolation Kit (Life Technologies, Cat. 88281) following manufacture's instruction. In brief, neonatal rat hearts were collected within 3 days after birth. After washing with ice cold HBSS [Hanks' Balanced Salt Solution (HBSS without Ca2+/Mg2+)], hearts are cut into small pieces before enzymatic digestion using Papain and Thermolysin for 35 min. After digestion, pieces are washed with cold HBSS once again and disassociated with cardiomyocyte culture medium with 10% FBS and single cells generated by filtrating over a 40 μm filter to remove undigested tissue. After centrifugation, cells were resuspended in culture medium with 10% FBS and seeded at 2.5 × 105 cells/cm^2^. The next day, cells were transfected with microRNA/siRNAs using RNAi Maxi (Invitrogen) following the manufacturer's instructions. Six hours after transfection, the medium was replaced with fresh DMEM medium with 10% FBS containing 1× Cardiomyocyte Growth Supplement. Twenty-four hours after transfection cells were either treated with indicated combinations of BrefeldinA (Sigma #B7651) 0.5 μg/ml, Salubrinal (Tocris #2347) 10 μM or vehicle. Forty-eight hours after transfection, cells were lysed for western blot analysis or fixed with 4% PFA for 15 min before TUNEL or GRP-78 staining.

### Western Blot Analysis

Cells or tissue was lysed with EDTA-free lysis buffer (Roche Applied Science, Cat.04719946001) with 1 × protease/phosphatase inhibitor cocktail (Cell Signaling, #5872). Protein concentrations were measured with BCA protein assay kit (Thermo Scientific, 23227), separated with NuPAGE bis-tris Precast gels (Life technologies), and transferred to PVDF membrane with an iblot Western blotting system (Life Technologies) according to the manufacturer's instructions. Membranes were blocked using 5% blotting grade blocker (Bio-Rad #170-6404). Antibodies used included polyclonal anti-ADAR1 (1:1,000; Abcam), anti-CHOP (1:1,000; Abcam ab170379), monoclonal anti-GAPDH (1:3,000; Chemicon), anti-IRE-1alpha (1:1,000; Abcam ab37073), followed by corresponding horseradish peroxidase (HRP)-conjugated secondary antibodies (1:2,500) and ECL detection (Sigma).

### Histological Analysis and (Immunofluorescence) Microscopy

Prior to histological analysis, hearts were arrested in diastole and prefusion-fixed with 4% paraformaldehyde (PFA), followed by paraffin embedding and sectioning at 4 μm. These sections were cut and mounted on coated slides and dried overnight at 37°C. Slides were dewaxed in xylene and hydrated using graded alcohols to tap water. The sections were consequently stained with FITC-labeled wheat-germ-agglutinin (WGA; Sigma) to visualize and quantify the cell cross-sectional area; haematoxylin and eosin (H&E; Sigma) for routine histological analysis; and Sirius Red (Sigma) for detection of fibrillar collagen. For immunofluorescence; sections were dried, fixed with 4% PFA, and permeabilized with 0.1% Triton X-100 (Sigma), dissolved in 1% BSA in PBS for 10 min and blocked with 10% goat serum in PBS for 60 min. Then, the slides were incubated o/n with primary antibody diluted in 0.1% BSA in PBS at 4°C. Secondary antibody incubation was performed at room temperature for 1 h, followed by 5 min Hoechst incubation and mounting with Fluoromount (Southern Biotech). Images were taken by a blinded investigator using the Olympus BX63 and cellSens and analyzed in ImageJ.

### GRP-78 Immunohistochemistry

Immunohistochemistry for GRP-78 was performed on sectioned paraffin-embedded tissue that was deparaffinized and heat-treated for antigen retrieval before staining. In brief, endogenous peroxidases were quenched with 3% hydrogen peroxide, endogenous biotin was blocked (Dako biotin blocking system; Dako, Carpinteria, CA), and non-specific staining was blocked with normal goat serum. Sections were incubated with primary antibody GRP-78 (Abcam, ab21685) overnight at 4°C, followed by host-specific biotin-conjugated secondary antibody. R&D Systems Streptavidin HRP was used for signal amplification prior to 15 min of incubation with Dako Liquid Permanent Red and embedding with Merck Millipore Entellan®. Images were taken by a blinded investigator using the Olympus BX63 and cellSens. The integrated optical density (IOD) was calculated using Image-Pro Premier. The intensity of immuno-reactivity of each sample was reflected by the mean values of the IOD.

### Tunel

TUNEL assays were performed on 10-μm frozen, apical cross-sections of hearts. Cryotome-cut frozen sections were first dried for 10 min at room temperature and fixed for 10 min in 4% PFA. Sections were subsequently used for TUNEL staining (*In situ* Death Detection Kit, Cat. 1684795, Roche) following manufacturer's instructions. After TUNEL, primary antibody anti-α-Actinin (Sigma-Aldrich, A7811) was applied overnight at 4°C with a concentration of 1:800. Secondary antibody incubation was performed at room temperature for 1 h, followed by 5 min Hoechst incubation and mounting with Fluoromount (Southern Biotech). Images were taken by a blinded investigator using the Olympus BX63 and cellSens and analyzed in ImageJ.

### Casp3/7 Assay

Following the manufacturer's instructions, heart tissue was lysed with EDTA-free lysis buffer (Roche Applied Science, Cat.04719946001) and protein concentrations were measured with BCA protein assay kit (Thermo Scientific, 23227). Fifty microliter of cell lysates were added in black 96 viewplates and 50 μl of homogenous Caspase-3/7 reagent (Apo-ONE® Homogeneous Caspase-3/7 Assay, Promega #G7790). The plate was incubated for 1 h at room temperature using a plate shaker at 350 rpm. After this time, fluorescence was measured in a fluorometer (Gemini, Molecular Devices) at excitation 499 nm and emission 521 nm.

### Statistical Analysis

The results are presented as mean ± standard error of the mean (s.e.m). All statistical analyses were performed with Prism software (GraphPad Software) consisting of ANOVA test followed by Tukey's post-test when group differences were detected at the 5% significance level, or Student's *t*-test when comparing two experimental groups. One-sided log-rank test was used for the Kaplan-Meier survival curve. Differences were considered significant when *P* < 0.05.

## Data Availability Statement

The raw data supporting the conclusions of this article will be made available by the authors, without undue reservation, to any qualified researcher.

## Ethics Statement

All experiments were carried out in accordance with the Guide for the Care and Use of Laboratory Animals, with prior approval by the Animal Ethical Experimentation Committee, Utrecht University.

## Author Contributions

HA, AV, and DF performed experiments. WG, PD, and RW provided reagents and discussed the study. HA, AV, DF, and JS analyzed data and designed the study. HA and JS wrote the manuscript.

### Conflict of Interest

The authors declare that the research was conducted in the absence of any commercial or financial relationships that could be construed as a potential conflict of interest.

## Supplementary Material

Supplemental Figure 1 **(A)** Real-time PCR analysis of transcript abundance of ADAR1 in mice hearts that were subjected to transverse aortic constriction for the indicated week(s). **(B)** Immunohistochemical staining of ER-stress associated protein GRP-78 in LAD infarcted heart depicting the ischemic and the remote region. **(C)** Western blot analysis of endogenous CHOP and GAPDH in neonatal rat cardiomyocytes transfected with miR-199a-5p mimicks and ADAR1 siRNA or scrambled miR for 48 h and treated with vehicle or BrefeldinA for 24 h. **(D)** Western blot analysis of endogenous CHOP and GAPDH in neonatal rat cardiomyocytes co-transfected with several candidate miRNAs and ADAR1 siRNA or scrambled miR for 48 h and treated with vehicle or BrefeldinA for 24 h. **(E)** Light microscopy images of neonatal rat cardiomyocytes co-transfected with several candidate miRNAs and ADAR1 siRNA or scrambled miR for 48 h and treated with vehicle or BrefeldinA for 24 h.

Supplemental Table 1 Echocardiographic analysis of αMHC-MCM-ADAR1^F/F^ and ADAR1^F/F^ mice, with/without tamoxifen treatment.

Supplemental Table 2 Echocardiographic analysis of αMHC-MCM-ADAR1^F/F^ treated with vehicle or Salubrinal and with/without tamoxifen treatment.
