# Supplementary figures and images for: Cardiomyocyte Specific Deletion of ADAR1 Causes Severe Cardiac Dysfunction and Increased Lethality
Source: Front Cardiovasc Med. 2020 Mar 18;7:30. doi: 10.3389/fcvm.2020.00030 (PMC7093378; doi:10.3389/fcvm.2020.00030)

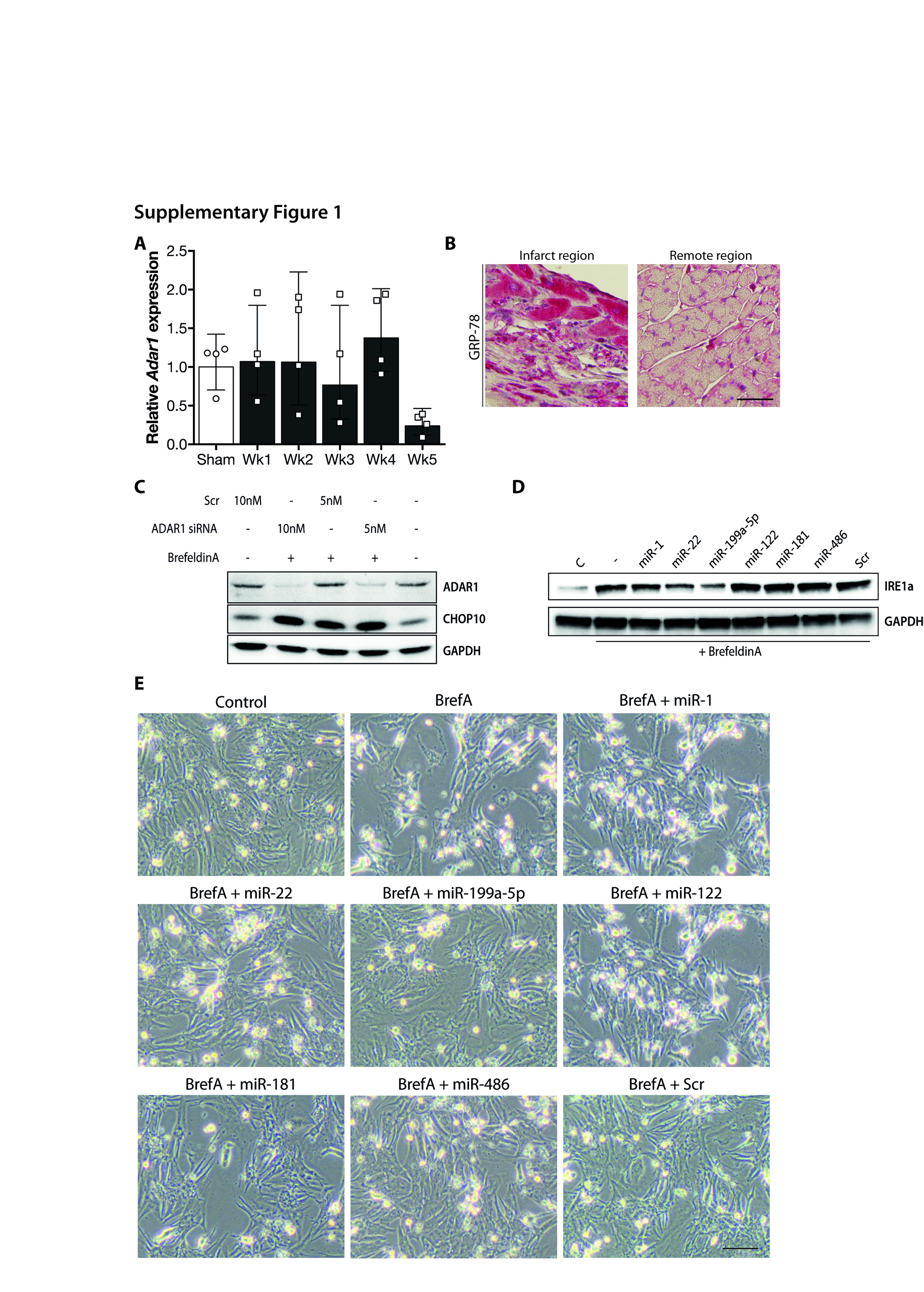

Supplement: Supplemental Figure 1 — (A) Real-time PCR analysis of transcript abundance of ADAR1 in mice hearts that were subjected to transverse aortic constriction for the indicated week(s). (B) Immunohistochemical staining of ER-stress associated protein GRP-78 in LAD infarcted heart depicting the ischemic and the remote region. (C) Western blot analysis of endogenous CHOP and GAPDH in neonatal rat cardiomyocytes transfected with miR-199a-5p mimicks and ADAR1 siRNA or scrambled miR for 48 h and treated with vehicle or BrefeldinA for 24 h. (D) Western blot analysis of endogenous CHOP and GAPDH in neonatal rat cardiomyocytes co-transfected with several candidate miRNAs and ADAR1 siRNA or scrambled miR for 48 h and treated with vehicle or BrefeldinA for 24 h. (E) Light microscopy images of neonatal rat cardiomyocytes co-transfected with several candidate miRNAs and ADAR1 siRNA or scrambled miR for 48 h and treated with vehicle or BrefeldinA for 24 h. [file Image_1.TIF]
