# Supplementary material for: Cardiomyocyte Specific Deletion of ADAR1 Causes Severe Cardiac Dysfunction and Increased Lethality
Source: Front Cardiovasc Med. 2020 Mar 18;7:30. doi: 10.3389/fcvm.2020.00030 (PMC7093378; doi:10.3389/fcvm.2020.00030)
Supplement: Supplemental Table 1 — Echocardiographic analysis of αMHC-MCM-ADAR1F/F and ADAR1F/F mice, with/without tamoxifen treatment. [file Table_1.DOCX]

|  | **MHC-MerCreMer-*ADAR1^F/F^*** | | ***ADAR1^F/F^*** |
| --- | --- | --- | --- |
|  | **Vehicle** | **Tamoxifen** | **Tamoxifen** |
| **N**  **Heart Rate** | 6  364 ± 29.7 | 9  381 ± 12.7 | 9  341 ± 28.4 |
| **LVVs (µl)** | 27.7 ± 3.4 | 60.8 ± 4.9 * | 28.7 ± 2.3 |
| **LVVd (µl)** | 69.8 ± 5.2 | 85.9 ± 5.3 * | 64.3 ± 4.2 |
| **LVIDs (mm)** | 2.51 ± 0.14 | 3.7 ± 0.12 * | 2.42 ± 0.12 |
| **LVIDd (mm)** | 3.97 ± 0.12 | 4.36 ± 0.11 * | 3.87 ± 0.10 |
| **SV (µl)** | 43.2 ± 2.76 | 25.4 ± 1.34 * | 42.3 ± 1.9 |
| **EF (%)** | 63.17 ± 1.42 | 30 ± 1.74 * | 67.5 ± 2.71 |
| **FS (%)** | 35.8 ± 1.45 | 13.7 ± 0.89 * | 35.17 ± 1.50 |

Table 1. Echocardiographic analysis of αMHC-MCM-ADAR1^F/F^ and ADAR1^F/F^ mice, with/without tamoxifen treatment.

Data are expressed as means ± SEM. LVVs, left ventricular volume at end-systole; LVVd, left ventricular volume at end-diastole; LVIDs, left ventricular internal dimension at end-systole; LVIDd, left ventricular internal dimension at end-diastole; SV, stroke volume; FS, fractional shortening.*, indicates *P*<0.05 vs vehicle treated αMHC-MCM-ADAR1^F/F^ group.
