# Supplementary material for: Cardiomyocyte Specific Deletion of ADAR1 Causes Severe Cardiac Dysfunction and Increased Lethality
Source: Front Cardiovasc Med. 2020 Mar 18;7:30. doi: 10.3389/fcvm.2020.00030 (PMC7093378; doi:10.3389/fcvm.2020.00030)
Supplement: Supplemental Table 2 — Echocardiographic analysis of αMHC-MCM-ADAR1F/F treated with vehicle or Salubrinal and with/without tamoxifen treatment. [file Table_2.docx]

Table 2. Echocardiographic analysis of αMHC-MCM-ADAR1^F/F^ treated with vehicle or Salubrinal and with/without tamoxifen treatment.

|  | **MHC-MerCreMer-*ADAR1^F/F^*** | | | |
| --- | --- | --- | --- | --- |
|  | **Salubrinal** | **Tamoxifen** | **Salubrinal/Tamoxifen** | |
| **N**  **Heart Rate** | 6  337.7 ± 10.3 | 8  336.9 ± 20.1 | 9  331 ± 16.1 * |  |
| **LVVs (µl)** | 28.5 ± 1.7 | 62.0 ± 7.3 | 40.4 ± 3.78 * |  |
| **LVVd ( µl )** | 55 ± 3.2 | 87.2 ± 6.9 | 79.6 ± 4.8 * |  |
| **LVIDs (mm)** | 2.3 ± 0.08 | 3.8 ± 0.18 | 3.1 ± 0.13 * |  |
| **LVIDd (mm)** | 4.0 ± 0.05 | 4.4 ± 0.14 | 4.2 ± 0.10 * |  |
| **SV (µl)** | 36.7 ± 2.7 | 25.3 ± 1.8 | 40.3 ± 1.9 * |  |
| **EF (%)** | 67.83 ± 1.19 | 30.33 ± 2.71 | 51.3 ± 2.94 * |  |
| **FS (%)** | 35.2 ± 1.17 | 14.2 ± 1.34 | 25.1 ± 1.76 * |  |

Data are expressed as means ± SEM. LV, left ventricular; IVSd, interventricular septal thickness at end-diastole; IVSs, interventricular septal thickness at end-systole; LVIDd, left ventricular internal dimension at end-diastole; LVIDs, left ventricular internal dimension at end-systole; LVPWd, left ventricular posterior wall thickness at end-diastole; LVPWs, left ventricular posterior wall thickness at end-systole; FS, fractional shortening.*, indicates *P*<0.05 vs vehicle treated αMHC-MCM-ADAR1^F/F^ group.
